# A systematic review of the efficacy and safety of anticoagulants in advanced chronic kidney disease

**DOI:** 10.1007/s40620-022-01413-x

**Published:** 2022-08-25

**Authors:** Kathrine Parker, John Hartemink, Ananya Saha, Roshni Mitra, Penny Lewis, Albert Power, Satarupa Choudhuri, Sandip Mitra, Jecko Thachil

**Affiliations:** 1 Manchester Institute of Nephrology and Transplantation, Manchester University NHS Foundation Trust, Oxford Road, Manchester, M13 9WL UK; 2 Division of Pharmacy and Optometry, School of Health Sciences, The University of Manchester, Manchester Academic Health Science Centre, University of Manchester, Manchester, M13 9PT UK; 3 Northern Care Alliance NHS Foundation Trust, Mayo Building, Salford Royal, Stott Lane, Salford, M6 8HD UK; 4 Department of Nephrology, North Bristol NHS Foundation Trust, Southmead Hospital, Southmead Road, Westbury-on-Trym, Bristol, BS10 5NB UK; 5 Department of Haematology, Northern Care Alliance NHS Foundation Trust, Royal Oldham Hospital, Rochdale Rd, Oldham, OL1 2JH UK; 6 Division of Cardiovascular Sciences, School of Medical Sciences, The University of Manchester, Manchester, M13 9NT UK; 7 Department of Haematology, Manchester University NHS Foundation Trust, Oxford Road, Manchester, M13 9WL UK

**Keywords:** Anticoagulation, Atrial fibrillation, Thrombosis, CKD, Stroke

## Abstract

**Background:**

Patients with chronic kidney disease (CKD) have an increased risk of venous thromboembolism (VTE) and atrial fibrillation (AF). Anticoagulants have not been studied in randomised controlled trials with CrCl < 30 ml/min. The objective of this review was to identify the impact of different anticoagulant strategies in patients with advanced CKD including dialysis.

**Methods:**

We conducted a systematic review of randomized controlled trials and cohort studies, searching electronic databases from 1946 to 2022. Studies that evaluated both thrombotic and bleeding outcomes with anticoagulant use in CrCl < 50 ml/min were included.

**Results:**

Our initial search yielded 14,503 papers with 53 suitable for inclusion. RCTs comparing direct oral anticoagulants (DOACs) versus warfarin for patients with VTE and CrCl 30-50 ml/min found no difference in recurrent VTE events (RR 0.68(95% CI 0.42–1.11)) with reduced bleeding (RR 0.65 (95% CI 0.45–0.94)). Observational data in haemodialysis suggest lower risk of recurrent VTE and major bleeding with apixaban versus warfarin. Very few studies examining outcomes were available for therapeutic and prophylactic dose low molecular weight heparin for CrCl < 30 ml/min. Findings for patients with AF on dialysis were that warfarin or DOACs had a similar or higher risk of stroke compared to no anticoagulation. For patients with AF and CrCl < 30 ml/min not on dialysis, anticoagulation should be considered on an individual basis, with limited studies suggesting DOACs may have a preferable safety profile.

**Conclusion:**

Further studies are still required, some ongoing, in patients with advanced CKD (CrCl < 30 ml/min) to identify the safest and most effective treatment options for VTE and AF.

**Supplementary Information:**

The online version contains supplementary material available at 10.1007/s40620-022-01413-x.

## Introduction

Patients with chronic kidney disease have an increased risk of venous thromboembolism (VTE), which includes both deep vein thrombosis (DVT) and pulmonary embolism. Patients with chronic kidney disease (CKD) stage 3 and 4 (eGFR 30–60 and eGFR15-29 ml/min/1.73 m^2^) have an adjusted relative risk 1.71 (95% CI 1.18 to 2.49) of developing DVT compared to those with normal renal function [1]. The risk is further heightened in those on dialysis where the age-adjusted pulmonary embolism incidence ratio is 2.34 compared to those with normal renal function [2]. With hospital-acquired VTE being a potentially preventable cause of in-hospital mortality the increased risk of VTE also highlights the need for appropriate VTE prophylaxis in a CKD population. Patients with CKD are also at increased risk of developing atrial fibrillation (AF) which may predispose them to thromboembolic stroke (ischaemic stroke). The prevalence of AF in the general population is estimated at around 1–2% [3], this rate is reported to be much higher as renal function declines, with reports of up to 10–25% of patients on dialysis [4–9].

Conversely, bleeding risk is also increased in the CKD population. It has been shown that the frequency of cerebral haemorrhage is over ten times higher in CKD patients than in the non-renal failure population [10], with an unpublished analysis from the Fresenius Medical Care Research database showing that 2.7% of all deaths in dialysis patients were secondary to haemorrhage [11].

Vitamin K antagonists (VKA) have been the mainstay of therapy for VTE treatment and AF in CKD. However, vitamin K antagonists are not without concerns in this population with difficulties obtaining time in therapeutic range [12], regular monitoring requirements, potential risks of vascular calcification and the rare condition calciphylaxis [13]. The use of direct oral anticoagulants (DOACs) is currently on the rise for both VTE and AF, however, in more severe renal impairment, CrCl < 30 ml/min, they have not been studied in randomised controlled trials (RCTs). Low molecular weight heparins (LMWHs) have been widely used for the initial treatment of VTE and are the mainstay of therapy for VTE prophylaxis, however, they have not been well studied in subjects with severe renal impairment, CrCl < 30 ml/min.

The aim of this systematic review is to identify the impact of different anticoagulant strategies in patients with advanced CKD CrCl < 50 ml/min, in terms of thrombotic and bleeding outcomes. This review provides the most up-to-date literature for anticoagulant strategies in AF, VTE and VTE prophylaxis for the various stages of advanced CKD including dialysis.

## Materials and methods

### Information sources and search strategy

The protocol for this review has been published in the International Prospective Register of Systematic Reviews (https://www.crd.york.ac.uk/prospero/, registration number CRD42020219449).

The following databases were used to undertake the search: Ovid MEDLINE (1946 to May 23, 2022), Embase (1974 to 2022 May 23), EBM Reviews—Cochrane Database of Systematic Reviews (2005 to May 18, 2022). Review papers were screened to identify any other relevant studies that had not been identified in the search. The search strategy was supported by a specialist librarian at the University of Manchester and was developed using MeSH terms and keywords relating to current anticoagulants in use. The full search strategy is detailed in supplementary appendix 1.

### Study selection

Randomised controlled studies and prospective or retrospective observational cohort studies were included. Included studies related to advanced CKD defined as CrCl or eGFR < 50 ml/min. Participants had either AF, VTE or required VTE prophylaxis. Studies that involved children < 18 years were excluded along with studies solely examining the use of anticoagulation to prevent clotting of the extracorporeal circuit. Other papers such as case studies, editorials, review articles, non-English studies and guidelines were excluded, although review papers were screened for relevant studies.

Only studies that reported both thrombotic and bleeding outcomes in patients taking an anticoagulant versus another anticoagulant or no anticoagulant were included.

### Data extraction

KP and AS screened the search results with any uncertainties being discussed with SM, PL and JT. At least two among KP, AS, RM and JH independently undertook data extraction into a modified version of the Cochrane data collection form. Extracted data included relevant baseline characteristics e.g. age, renal function, thrombotic and bleeding risk factors, methods (study design, duration of follow up), interventions (treatment and comparator) and outcomes. Efficacy outcomes included the incidence of stroke and systemic embolism for AF and incidence of VTE/VTE recurrence in the treatment or prophylaxis of VTE. Safety outcomes included the incidence and severity of bleeding episodes.

### Risk of bias

For randomised controlled studies the revised Cochrane Risk-Of-Bias tool (ROB 2) was used [14]. For non-randomised studies the Risk Of Bias In Non-randomized Studies – of Interventions (ROBINS-I) tool was used [15]. KP, AS, RM and JH independently undertook bias assessment to determine study quality with disagreement being resolved by PL.

## Results

### Study yield

**Fig. 1:**
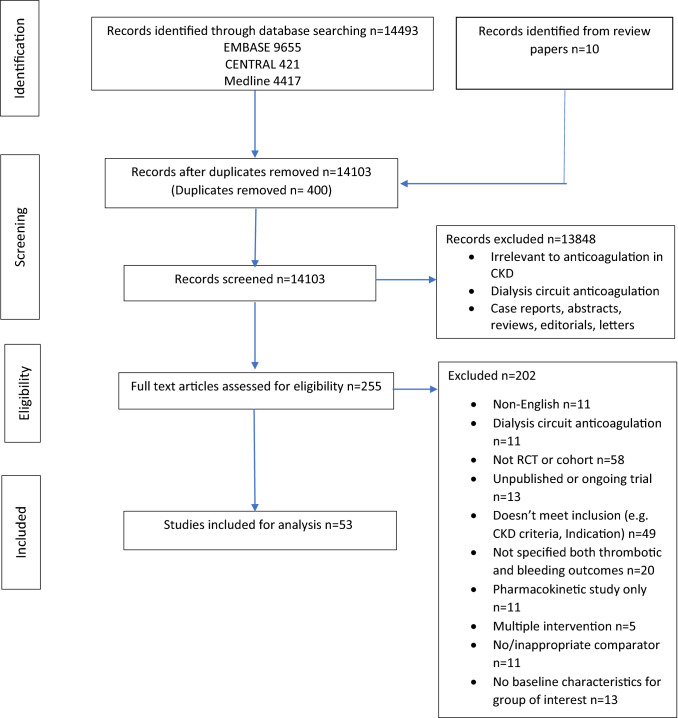
Flow diagram of study selection

Our initial search yielded 14,503 papers, of which 14,103 were screened after duplicates were removed. Another 13,848 were removed due to being irrelevant to anticoagulant use in CKD, related only to dialysis circuit anticoagulation or being an editorial, case report, review or guideline. There were 255 papers that included anticoagulant use in CKD, and after further assessment, 53 papers were suitable for inclusion. The overall study selection process is detailed in Fig. 1.

**VTE treatment in patients with CrCl < 50 ml/min**

### Study characteristics

An overview of the selected VTE studies is presented in Table 1. There were four RCTs of DOACs versus VKA, of which two were analyses of the CKD subgroup [16–19]. One cohort study was included that studied cancer-associated VTE [20]. These studies focused mainly on patients with CrCl 30–50 ml/min, however, there were small numbers of patients with a CrCl < 30 ml/min in two of the RCTs and within the cohort study [16, 18, 20]. All of the RCTs used Cockcroft-Gault creatinine clearance and the cohort study used Modified Diet in Renal Disease equation  [21]. An observational study in dialysis patients is also included which compared apixaban to warfarin in the acute treatment of VTE [22].

**Table 1:** Characteristics and outcomes of included VTE treatment studies

Study	Study design	Follow up	Renal function	Treatment (*n*)	Comparator (*n*)	Age, years	Risk factors	Outcome measures	Recurrent VTE treatment vs comparator	HR recurrent VTE	Bleeding Treatment vs comparator	HR bleeding
Agnelli 2013 [18] AMPLIFY	RCT Double-Blind	7 months	CrCl ml/min > 30– < 50 < 30	Apixaban 10 mg bd for 7 days then 5 mg bd *n* = 161 *n* = 14	Enoxaparin/VKA *n* = 148 *n* = 15	Mean (SD) Apixaban 57.2 (16) VKA 56.7 (16)	Previous VTE 17.2 vs 15.1% Cancer 2.5% vs 2.8%	Composite of fatal or nonfatal PE or DVT Major bleeding as defined by ISTH	7 vs 7	NR	5 vs 9	NR
Bauersachs 2014 [16] EINSTEIN PE and DVT	RCT Open-Label	Rivaroxaban 263 days Warfarin 268 days	CrCl ml/min 30–49 < 30	Rivaroxaban 15 mg bd for 3 weeks then 20 mg od *n* = 323 *n* = 10	Enoxaprin/VKA *n* = 313 *n* = 11	Median (Q1-Q3) Rivaroxaban 80.0 (75.0–84.0) VKA 79.0 (75.0–83.0)	Previous VTE 19.1% vs 19.8% Cancer 5.6% vs 4.8%	Composite of fatal or nonfatal PE or DVT Major bleeding as defined by ISTH Composite of Major and CRNMB	11 vs 10 0 vs 1	HR 1.05 (95% CI 0.44–2.47) n/a	Major: 30-49 ml/min 3 vs 12 < 30 ml/min 0 vs 1 Composite 37 vs 43 2 vs 1	Major bleeding0.23 (95%CI 0.06–0.81) n/a
Buller 2013 [17] Hokusai-VTE	RCT Double-Blind	12 months	CrCl ml/min 30–50	Edoxaban 30 mg od following 7 days of LMWH therapy *n* = 268	LMWH/VKA 273	Mean (SD) Edoxaban 55.7 (16.3) VKA 55.9 (16.2)	Previous VTE 19% vs 17.9% Cancer 9.2% vs 9.5%	Composite of fatal or nonfatal PE or DVT Composite of major and CRNMB as defined by ISTH	8 vs 16	NR	28 vs 39	NR
Goldhaber 2013 [19] Pooled analysis of RECOVER I and II	RCT Double-Blind	Dabigatran 163 days Warfarin 163 days	CrCl ml/min 30–50	Dabigatran 150 mg bd *n* = 114	Warfarin 123	Mean (SD) Dabigatran 54.8 (16) VKA 54.7 (16.2)	Not stated	Composite of fatal or nonfatal PE or DVT Major bleeding as defined by ISTH Composite of major/ CRNMB	0 vs 5	NR	6 vs 5 Composite 12 vs 12	NR
Kooiman 2013 [20]	Retrospective cohort RIETE and Dutch registries	LMWH 91 (4–178) VKA 156 (2–180)	eGFR MDRD ml/min/1.73m^2^ 30–45 < 30	VKA *n* = 99 *n* = 51	LMWH (unspecified) *n* = 151 *n* = 67	Mean (SD) 74.6(10.9) < 30 73.8(13.2)	Cancer-related VTE in all patients	Fatal or non-fatal PE (including PE at autopsy) and objectively verified DVT Major bleeding defined as per ISTH Fatal bleeding	1 vs 12 2 vs 1	VKA vs LMWH aHR 0.1 (0–0.8) eGFR < 30 n/a	5 vs 7 2 vs 13 Fatal 1 vs 4 0 vs 6	eGFR 30–45 VKA vs LMWH aHR 2.4 (0.6–9.4) eGFR < 30 VKA vs LMWH aHR 0.5 (95% CI 0.1–2.8)
Wetmore 2022 [22]	Retrospective cohort	6 months or until outcome of interest	Dialysis	VKA 9086	Apixaban 3130 Dose not specified but the authors assumption is that the FDA licensed dose 10 mg bd for 7/7 then 5 mg bd has been used (personal correspondence James Wetmore)	Age (%) 18–44 13% vs 13% 45–64 37% vs 37% 65–74 28% vs 28 75–79 10% vs 10% 80 + 12% vs 12%	Cancer 14% vs 14% Hospital stay ≥ 3 days 52% vs 53% Surgery 24% vs 24%	Recurrent VTE Major bleeding including fatal bleeding, critical site bleeding or required a blood transfusion	8.3 per 100 patient years Vs 4.9 per 100 patient years	HR 0.58 (95% CI 0.43- 0.77)	11.1 per 100 patient years vs 8.8 per 100 patient years	HR 0.78 (95% CI 0.62–0.98)

Randomised controlled trial (RCT), Cockcroft-Gault Creatinine Clearance (CrCl), Vitamin K antagonist (VKA), Venous thromboembolism (VTE), Low molecular weight heparin (LMWH), MDRD (modified diet in renal disease), once daily (od), twice daily (bd), Hazard Ratio (HR), Not reported (NR), Pulmonary embolism (PE), Deep Vein Thrombosis (DVT), Confidence Interval (CI), International Society on thrombosis and haemostasis (ISTH), Clinically relevant non-major bleeding (CRNMB), adjusted Hazard Ratio (aHR)

### Quality assessment

Assessment of the quality of the four RCTs found the studies to be of high quality and of low risk of bias [16–19]. The two cohort studies were deemed at low [22] or moderate risk of bias [20], Supplementary Tables 1 and 2.

#### Recurrent VTE outcomes

Six studies reported on recurrent VTE during treatment of VTE. An overview of these outcomes is presented in Table 1.

##### DOACs vs warfarin

**Fig. 2:**
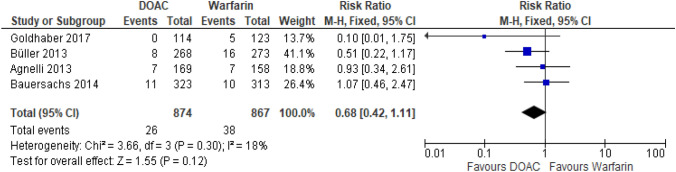
Recurrent VTE events from RCTs in patients with CrCl 30-50 ml/min [16–19]. Figure created using RevMan software [23]. Please note the number of patients from Agnelli 2013 in this analysis differs from the total number of patients in Table 1. The numbers are taken directly from the paper and it is assumed the difference relates to missing outcome data or loss of follow up [18]

There were four RCTs comparing DOACs to warfarin for the treatment of VTE [16–19]. There was no significant difference between the efficacy of the DOACs compared to warfarin for patients with CrCl 30-50 ml/min although there was a trend for increased efficacy of dabigatran [19], Fig. 2. The cohort study found apixaban had a reduction in recurrent VTE compared to warfarin in a haemodialysis population being treated for acute VTE (HR 0.58 (95%CI 0.43–0.77)) [22].

##### LMWH vs warfarin

One retrospective cohort study examined patients treated for cancer-related VTE [20]. For those with an eGFR 30–45, warfarin was associated with less recurrent VTE than LMWH, 2.9/100 person years versus 25.5/100 person years [20]. The major limitation to this study is that the type of LMWH and dose used was not reported and a number of patients (285/1684) switched between treatment regimens [20].

#### Major bleeding

An overview of the definition and incidences of bleeding is presented in Table 1.

##### DOACs vs warfarin

**Fig. 3:**
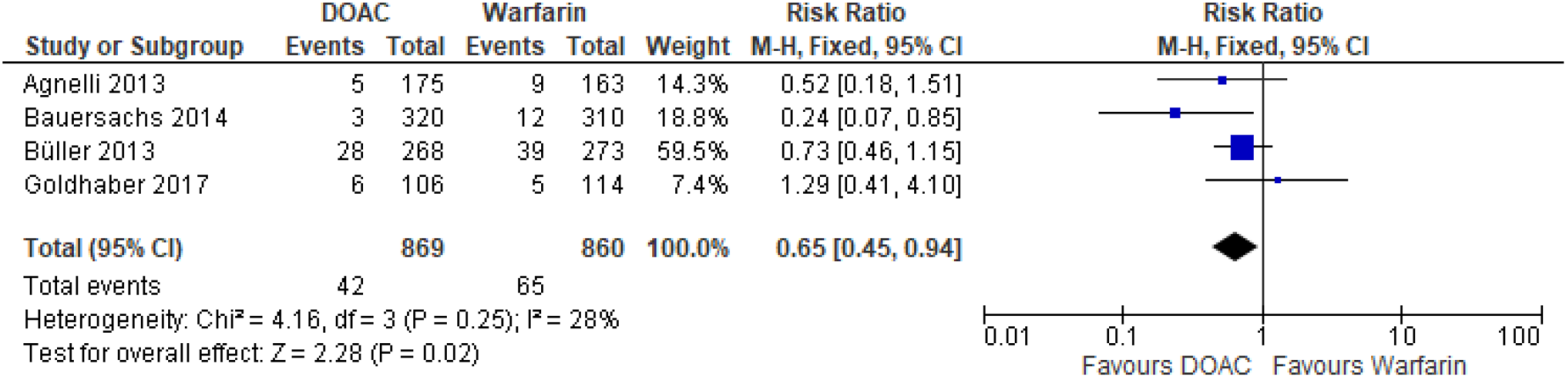
Major bleeding events from RCTs in patients with CrCl 30-50 ml/min [16–19]. Note that the Buller study is a composite outcome of major bleeding and CRNMB. Figure created using RevMan software [23]. Please note the number of patients from Bauersachs 2014 and Goldhaber 2017 in this analysis differs from the total included patients in Table 1. The numbers are taken directly from the papers and it is assumed the difference relates to missing outcome data or loss of follow up [16, 19]

The RCT sub-analyses looking at apixaban and rivaroxaban found lower rates of major bleeding in patients with CrCl 30–49 ml/min versus warfarin [16, 18]. For apixaban, major bleeding was 2.9% vs 5.5% in the warfarin arm [18] and rivaroxaban 0.9% vs 3.9% in the warfarin arm [16]. The major bleeding rates for dabigatran were similar to warfarin [19]. Major bleeding of the DOACs versus warfarin is presented in Fig. 3. The cohort study found that for patients on haemodialysis apixaban use was associated with a HR 0.78 (95% CI 0.62–0.98).

##### LMWH vs warfarin

The cohort study found that LMWH was associated with both higher major and fatal bleeding in patients with GFR < 30 ml/min, VKA vs LMWH, aHR 0.5 (95% CI 0.1–2.8) [20], although these findings should be interpreted with caution given the wide confidence intervals and lower interval close to no difference. In subjects with CrCl 30–49 ml/min there was no real difference in bleeding events [20].

#### VTE Prophylaxis in non-surgical patients with CrCl < 50 ml/min

Two cohort studies met inclusion criteria, both in the haemodialysis population [24, 25], Supplementary Table 3. These studies examined VTE prophylaxis for patients with an acute medical illness or a recent hospital admission with ongoing risk factors for VTE. Both studies used enoxaparin compared to unfractionated heparin (UFH); one study used the FDA licensed dose of enoxaparin 30 mg daily [25], whilst the second study had variable enoxaparin dosing ranging from 20 mg daily to 60 mg daily [24]. The heparin dose remained consistent in the first cohort study at 5000units three times daily [25] with doses ranging from 5000units twice to three times daily in the second study [24].

Quality

The largest cohort study was deemed to have moderate bias due to potential selection bias and varying doses of both treatments [24]. The second cohort study was deemed to have a serious/critical bias as it did not use any statistical methods to take into consideration confounders between the treatment and comparator groups [25], Supplementary Table 2.

##### Venous thromboembolism outcomes with VTE prophylaxis in patients with CrCl < 50 ml/min

An overview of VTE outcomes is presented in Supplementary Table 3.

*Enoxaparin versus subcutaneous unfractionated heparin*

The short follow up period in one of the cohort studies meant no VTE events were seen in either group [25]. The second cohort study examined a much more prolonged period of prophylaxis and event rates were the same between the enoxaparin and UFH groups, 2.7 per 100 patient years [24].

##### Major bleeding outcomes with VTE prophylaxis in patients with CrCl < 50 ml/min

The details of major bleeding outcomes are reported in Supplementary Table 3.

*Enoxaparin versus subcutaneous unfractionated heparin*

With the short follow up in one cohort study no major or clinically relevant non major bleeding were reported in either group [25]. In the second cohort study there was no difference in major bleeding between the groups, UFH 17.2 bleeds per 100 patient years vs enoxaparin 16.9 per 100 patient years [24], *p* = 0.02 [24] for equivalence.

#### Anticoagulant treatment in patients with AF and CrCl < 50 ml/min

Study characteristics

There were 45 studies that included anticoagulant use in patients with CrCl < 50 ml/min, five RCTs and 40 cohort studies. An overview is provided in Supplementary Table 4. Due to the heterogeneity in the way bleeding and stroke outcomes were reported, alongside variations in renal function and varying doses of anticoagulants, a meta-analysis was not conducted.

Quality

Four of the RCTs were found to be of high quality and of low risk of bias and one RCT showed some concerns relating to it being powered to detect an alternative outcome. Of the 40 cohort studies twelve were deemed of good quality with low bias, nineteen were deemed to be of moderate quality, whilst the remaining nine had severe risk of bias, Supplementary Tables 1 and 2.

##### Stroke outcomes in studies comparing DOACs to warfarin in patients with AF and CrCl < 50 ml/min

An overview of stroke outcomes is presented in Supplementary Table 5.

**Fig. 4:**
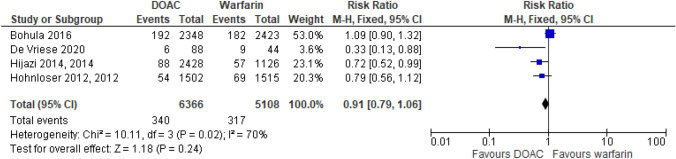
Stroke reported from RCTs in patients with AF and CrCl < 50 ml/min. Figure created using RevMan software [23]

Four RCT sub-analyses looking at DOACs versus warfarin included patients with CrCl 30–50 ml/min (25–50 ml/min for apixaban) [26–29]. They found the risk of stroke or systemic embolism (SSE) was similar between the DOACs and warfarin, Fig. 4. Four cohort studies examining patients with CrCl ≤ 45 ml/min found that rivaroxaban had a reduced risk of SSE compared to warfarin [30–33]. Doses of rivaroxaban used in these studies were variable and included 20 mg, 15 mg and 10 mg daily. A Japanese study by Koretsune et al. found that apixaban reduced the risk of SSE in patients with a CrCl 15–49 ml/min compared with warfarin [34]. In a haemodialysis population there were four studies comparing DOACs to warfarin [35–38]. A RCT studied rivaroxaban 10 mg daily in a haemodialysis population and found a reduction in SSE with rivaroxaban but this study was not powered to detect a difference in stroke [35]. The use of apixaban in two retrospective studies of dialysis patients found no significant reduction in stroke compared to those on warfarin, with patients taking apixaban 2.5 mg or 5 mg twice daily [36, 38]. Conversely, in a haemodialysis population, Chan et al. found that rivaroxaban and dabigatran had an increased risk of embolic stroke and arterial embolism compared to warfarin [37]. The remaining five studies in patients with CrCl < 60 ml/min found no difference in stroke between the DOAC and warfarin groups [39–43].

##### Stroke outcomes from studies comparing oral anticoagulation to no anticoagulation in patients with AF and CrCl < 50 ml/min

Of the 27 studies looking at oral anticoagulation versus no anticoagulation, 23 were based in a dialysis population and the remaining four studies in patients with eGFR < 60 ml/min/1.73 m^2^, Supplementary Table 5.

Two studies in patients with CrCl < 60 ml/min found a benefit from warfarin compared to no anticoagulation [44, 45]. The third study in patients with eGFR < 30 ml/min/1.73 m^2^ found stroke rates were higher in those on DOACs or VKAs compared to no anticoagulation [46], whilst a fourth found a reduction in stroke with DOACs but not warfarin compared to no anticoagulation [47]. In a dialysis population, Mavrakanas et al. found no difference in the incidence of stroke when apixaban was compared to no treatment in a propensity matched dialysis cohort, HR1.24 (0.69–2.23) [48]. From a national Taiwan database See et al. also found no benefit of warfarin or DOACs in reducing the risk of ischaemic stroke in those on dialysis [49], whilst a large retrospective study by Agarwal et al. found that warfarin increased the risk of ischaemic stroke compared to no anticoagulation [50]. Of the remaining dialysis studies, five found benefit from warfarin in reducing the risk of ischaemic stroke [51–55], eleven studies found no benefit of warfarin in reducing the risk of stroke [5, 56–65] and four studies found warfarin led to an increased risk of stroke [66–69], Supplementary Table 5.

##### Major bleeding outcomes from studies comparing DOACs to warfarin in patients with AF and CrCl < 50 ml/min

An overview of major bleeding outcomes is presented in Supplementary Table 6.

**Fig. 5:**
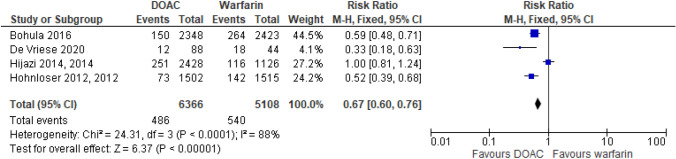
Major bleeding from RCTs in patients with AF and CrCl < 50 ml/min. Figure created using RevMan software [23]

From the RCTs Hohnloser et al. found that apixaban was associated with a reduced risk of major bleeding compared to warfarin in patients with a CrCl < 50 ml/min [29], Fig. 5. This was also seen with edoxaban [28], but not with rivaroxaban and dabigatran where bleeding was similar to warfarin [27]. Edoxaban was also found to have significant reduction in the risk of intracranial haemorrhage, HR 0.46 (95% CI 0.26–0.82) *p* = 0.009, in patients with CrCl 30–50 ml/min compared to warfarin [26, 28]. Similar findings of intracranial haemorrhage reduction were seen with dabigatran in CrCl < 50 ml/min for both 150 mg and 110 mg, respectively, HR 0.31 (95% CI 0.14–0.66) and 0.40 (95% CI 0.20–0.80) [27].

De Vriese et al. found a significant reduction in major bleeding with rivaroxaban compared to warfarin in haemodialysis patients, although the study was small [35]. Similar findings were seen by Siontis and Wetmore who both found a reduction in major bleeding with apixaban compared to warfarin in dialysis patients, HR 0.72 (95% CI 0.59–0.87) *p* < 0.001 [36] and HR 0.67 (95% CI 0.55–0.81) label dose, respectively [38]. Conversely, an observational study by Chan et al. in haemodialysis patients found that compared to warfarin rivaroxaban and dabigatran had higher rates of major bleeding including higher rates of death from bleeding [37]. Despite being on haemodialysis, 15.3% patients on dabigatran and 32.1% on rivaroxaban were still on non-renal adjusted doses [37], Supplementary Table 6.

The remaining ten observational studies were in patients with eGFR < 60 ml/min/1.73 m^2^. Six of these studies found DOACs had a lower risk of major bleeding compared to warfarin [30, 31, 34, 36, 39, 40], three found no difference [33, 42, 43], whilst a study by Shin et al. showed that rivaroxaban and dabigatran compared to warfarin had a higher risk of major bleeding [41], Supplementary Table 6.

##### Major bleeding outcomes from studies comparing OAC to no anticoagulation in patients with AF and CrCl < 50 ml/min

Of the 27 studies comparing oral anticoagulation versus no anticoagulation, 23 were based in a dialysis population and the remaining four studies in patients with eGFR < 60 ml/min/1.73 m^2^, Supplementary Table 6.

From the four studies in patients with eGFR < 60 ml/min/1.73 m^2^ there was less major bleeding in patients not taking anticoagulants, and the difference in major bleeding was more evident as renal function declined [44–47], supplementary table 6. Fourteen studies in dialysis patients found an increased risk of bleeding events with anticoagulation as compared to none [48, 50, 53, 54, 56–62, 65, 67, 69]. However, there were nine studies in dialysis patients that found no difference in major bleeding between those anticoagulated and not [5, 49, 51, 52, 55, 63, 64, 66, 68].

## Discussion

This is the first systematic review of VTE prophylaxis in patients with renal impairment that focuses on both thrombotic and bleeding outcomes for medical patients and the most up-to-date review of bleeding and thrombotic outcomes in the treatment of VTE and AF. This review includes the available evidence for anticoagulants across the various stages of CKD, Table 2, which highlights a distinct lack of studies that describe both thrombotic and bleeding outcomes in patients with a CrCl < 30 ml/min. Inclusion of multiple indications for anticoagulant use makes this review a holistic resource for nephrology professionals.

**Table 2:** Summary of evidence available for DOACs versus warfarin in varying stages of CKD in patients with VTE and AF

vs warfarin	eGFR 30–50 ml/min/1.73 m^2^	eGFR 15–30 ml/min/1.73 m^2^	eGFR < 15 ml/min/1.73 m^2^ not on dialysis	Dialysis
Treatment of VTE				
Rivaroxaban	Similar efficacy with lower rates of major bleeding [16]	No information	No information	No information
Dabigatran	Trend towards increased efficacy with no significant difference in major bleeding [19]	No information	No information	No information
Edoxaban	Similar efficacy with no significant difference in major bleeding [17]	No information	No information	No information
Apixaban	Similar efficacy with lower rates of major bleeding [18]	No information	No information	Reduced risk of recurrent VTE and major bleeding [22]. Dosing information is not reported but it is anticipated the majority took FDA licensed doses of 10 mg bd for 7 days then 5 mg bd (authors assumption personal correspondence)
Atrial fibrillation				
Rivaroxaban	Similar risk of SSE and major bleeding [26, 39, 43]. A reduced risk of intracranial haemorrhage has been shown	The majority of studies found similar rates of SSE and major bleeding with rivaroxaban with most patients receiving 15 mg daily [31, 32, 39, 42]	The majority of studies found similar rates of SSE and major bleeding with rivaroxaban with most patients receiving 15 mg daily [31, 32, 39]	One study found no difference in SSE with a reduced risk of major bleeding using 10 mg daily [35] A second study found an increased risk of embolic stroke and major bleeding with 15 mg or 20 mg daily [37] Other studies with limited numbers of dialysis patients found similar risk of SSE and major bleeding [31, 32]
Dabigatran	Dabigatran 150 mg bd had a trend for reduced risk of SSE whilst 110 mg bd had a similar risk of stroke. Risks of major bleeding are similar [27, 39–41]	Studies show similar risks of SSE and major bleeding [39, 41] although outcomes presented may be combined with other DOACs	Studies show similar risks of SSE and major bleeding [39, 40], although outcomes presented may be combined with other DOACs	One study showed an increased rate of embolic stroke and major bleeding [37]
Edoxaban	Edoxaban had a similar risk SSE with reduced risk of major bleeding and intracranial haemorrhage [28]	No information	No Information	No information
Apixaban	Apixaban has been shown to have similar rates of SSE with a reduced risk of major bleeding [29, 39–41] In a Japanese study a reduction in stroke and major bleeding was seen [34]	The majority of studies show apixaban has similar rates of SSE with a reduced risk of major bleeding [39–41] In a Japanese study a reduction in stroke and major bleeding was seen [34]	Apixaban has been shown to have a similar rate of SSE with a reduced risk of major bleeding [39, 40]	There is no difference in SSE with apixaban (2.5 mg bd or 5 mg bd) but there is a reduced risk of major bleeding [36, 38]

The first issue on which the review is focused regards the use of DOACs for patients with VTE and  CrCl <50 mL/min.  Four RCTs compared DOACs versus warfarin in patients with CrCl 30–50 ml/min with no difference in recurrent VTE events. Dabigatran had a trend towards increased efficacy but due to its high renal excretion it would not be the first choice in a CKD population. There was no significant difference in major bleeding between the DOACs and warfarin although apixaban and rivaroxaban had lower rates of major bleeding making them a suitable treatment option for VTE treatment in patients with CrCl ≥ 30 ml/min.

Overall findings from a recent systematic review showed DOACs had a reduced major bleeding risk compared to VKAs in CKD patients (this included patients with mild renal impairment) [70]. Cheung et al. compared DOACs with warfarin for VTE treatment in dialysis patients [71] and suggested apixaban as an option without a loading dose and a reduced maintenance dose. However, this review included studies reporting combined outcomes for patients with indications not limited to VTE, with the majority having AF [72–77], and studies failed to report VTE risk factors (Supplementary Table 7). Heterogeneity of apixaban dosing for VTE is also seen, particularly in terms of loading doses. Based on the more recent observational study from Wetmore et al. [22], where the majority of dialysis patients are believed to be taking the Food and Drug Administration licensed apixaban dose of 10 mg twice daily for a week followed by 5 mg twice daily (personal correspondence James Wetmore), this would support the use of higher doses in haemodialysis than that proposed by Cheung et al. [71]. The reduced risk of recurrent VTE and major bleeding shown by Wetmore and colleagues may in part be explained by the problems in achieving and maintaining INR target ranges with warfarin in a dialysis population [12].

An observational study comparing rivaroxaban to standard anticoagulation, [78], found that major bleeding was similar between rivaroxaban, warfarin and LMWH/fondaparinux. In terms of recurrent VTE, the findings were similar for rivaroxaban and warfarin with higher rates for those on LMWH/fondaparinux. Baseline characteristics were not available for CKD patients, thus selection bias is possible.

**Fig. 6:**
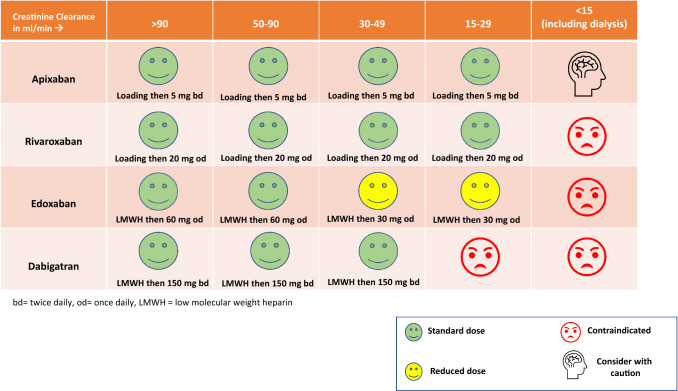
Authors suggested use of DOACs in patients with acute VTE depending on renal function

Anti-Xa agents are licensed for treating VTE in patients with CrCl 15–29 ml/min, based on limited clinical outcome data. Venous Thromboembolism in Renally Impaired Patients and Direct Oral Anticoagulants (VERDICT), is currently investigating reduced doses of apixaban and rivaroxaban for the treatment of VTE in patients with CrCl 15–50 ml/min compared to conventional therapy with LMWH and VKAs. At present there is still limited information about the use of DOACs for VTE treatment in those with CrCl < 15 ml/min but apixaban may be an option for those on dialysis, although the optimal dosing regimen is still unclear. The authors of this review present their suggestions for the use of DOACs in acute VTE based on renal function in Fig. 6.

The second issue on which the review is focused regards the use of LMWH in the treatment of VTE in patients with CrCl<50 mL/min.   Similar to the REMOTEV study [78], Kooiman et al. found LMWH was less effective in preventing recurrent VTE than warfarin in patients with cancer and CrCl 30–49 ml/min [20]. This study highlighted an increase in both major bleeding and fatal bleeding with LMWH in patients with a CrCl < 30 ml/min. The manufacturers of the LMWHs advise caution with their use in these populations due to potential accumulation [79–81].

No studies examining outcomes of therapeutic LMWH use in CKD met inclusion for this review. Pon et al. undertook a review of haemodialysis patients taking intravenous UFH or therapeutic subcutaneous (SC) enoxaparin finding that there was no difference in major bleeding or thromboembolic events [82]. Similarly, Thorevska 2003 found no difference in major bleeding between intravenous UFH and twice-daily enoxaparin in patients with CrCl < 20 ml/min [83]. A retrospective review of therapeutic UFH versus enoxaparin in patients with eGFR < 60 ml/min found UFH was associated with an increase in major bleeding, HR 4.79 [95% CI, 1.85–12.36] [84].

The Comparison of Acute Treatments in Cancer Haemostasis (CATCH) study which compared tinzaparin with warfarin in cancer-associated thrombosis (including those with an eGFR < 60 ml/min/1.73 m^2^ [85] found no difference in recurrent VTE or major bleeding. The Innohep® in Renal Insufficiency Study (IRIS) designed to look at bleeding and safety outcomes of tinzaparin vs therapeutic UFH SC in patients with VTE and CrCl < 30 ml/min was terminated early due to excess mortality within the tinzaparin group although r review has deemed this not related to bleeding or thrombotic events. A sub-study of the IRIS trial looking at accumulation of tinzaparin in 21 patients with CrCl < 30 ml/min (dialysis excluded) found no accumulation over 8 days when dosed at 175units/kg SC daily [86]. A further study also demonstrated no tinzaparin accumulation in patients with CrCl 20-34 ml/min for up to 30 days [87].

A study of therapeutic dalteparin compared to intravenous UFH in patients with an eGFR < 60 ml/min found a significant reduction in major bleeding in the dalteparin arm, even in those with eGFR < 30 ml/min [88]. Schmid et al. however found significant dalteparin accumulation in those with CrCl < 30 ml/min which led them to recommend anti-Xa monitoring in this population [89].

When using therapeutic doses of LMWH in patients with severe renal impairment, CrCl < 30 ml/min, anti-Xa level monitoring should be considered [79–81] with dose reductions as appropriate [90, 91]. The European Society of Cardiology (ESC) recommends that if LMWH is prescribed in patients with CrCl 15 − 30 mL/min, an adapted dosing scheme should be used [92].

The third issue regards VTE prophylaxis in non-surgical patients with CrCl <50 mL/min.   There were limited studies in this group. The two included studies both looked at enoxaparin versus UFH prophylaxis in haemodialysis patients. Green et al. showed short duration of prophylaxis and noted no thromboembolic or bleeding events in either group [25]. The much larger study by Chan et al. showed there was no difference in major bleeding rates between enoxaparin and UFH [24]. However, both studies used the FDA licensed dose (30 mg –daily) which differs from European dosing (20 mg or 40 mg). Chan et al. also used off-license dosing of up to 60 mg SC daily; however,indications were unclear, and 28% of patients were defined as obese [24]. Mahe examined accumulation of LWMH prophylaxis in elderly patients with CrCl 20–50 ml/min and lower bodyweight (mean 52 kg) [93]. After eight days, they found accumulation of enoxaparin but not tinzaparin [93].

A systematic review investigating anti-Xa monitoring of LMWHs for VTE prophylaxis in CKD patients concluded that prophylactic doses of dalteparin and tinzaparin did not accumulate, but enoxaparin did in those with CrCl < 30 ml/min, and thus a dose reduction would be required [94].

The fourth issue regards anticoagulant use in atrial fibrillation in patients on dialysis. The benefits of anticoagulation in reducing the risk of SSE in dialysis patients with AF are uncertain. The majority of studies found that warfarin or DOACs had either a similar risk of stroke or a higher risk compared to no anticoagulation. The heterogeneity in which stroke was defined in these studies and the difference in baseline population characteristics makes it difficult to compare these studies. In terms of safety outcomes, anticoagulation had a similar or elevated risk of major bleeding versus no anticoagulation. An increase in intracranial haemorrhage with the use of anticoagulation was a notable finding. Some studies reporting gastrointestinal (GI) bleeding found similar rates between those taking and not taking anticoagulation. This may be related to the pre-existing high rates of gastrointestinal bleeding [51, 52, 59, 67] that are observed in a dialysis population, independent of anticoagulant use [95, 96], which is mainly related to increased angiodysplasia [97].

For patients with AF and end-stage renal disease clinical equipoise exists where the risk of bleeding with any anticoagulant may outweigh any potential benefits [98–101]. To investigate whether anticoagulation is appropriate for dialysis patients with AF, the ongoing AVKDIAL, DANWARD, SACK and SAFE-D studies may provide some answers [102–105]. AVKDIAL and DANWARD are both open-label RCTs looking at vitamin K antagonist versus no anticoagulation on the risk of stroke and major bleeding [102, 104]. Whilst the Swedish SACK study is looking at apixaban 2.5 mg twice daily versus no treatment in patients with eGFR < 15 ml/min/1.73 m^2^, including dialysis, on stroke and bleeding outcomes [105]. The SAFE-D pilot trial aims to assess the feasibility of setting up a larger RCT in AF patients on dialysis [103]. This trial is currently randomising patients to one of three arms: warfarin, apixaban 2.5 mg bd or no anticoagulation.

We noted no advantage of DOACs in reducing the risk of SSE as compared to warfarin, with one study showing an increased risk of stroke with dabigatran and rivaroxaban [37]. In terms of major bleeding, Siontis et al. [36] and Wetmore et al. [38] showed a significant reduction in major bleeding with apixaban compared to warfarin as did De Vriese et al. [35] with rivaroxaban 10 mg daily. Conversely Chan et al. showed a significant increase in major bleeding, including fatal bleeding, with dabigatran and rivaroxaban 20 mg or 15 mg daily compared to warfarin [37]. US observational studies looking at DOACs versus warfarin in dialysis included multiple indications making it difficult to extract the patient outcomes (Supplementary Table 7).

Unfortunately, the RENAL-AF study, an open-label RCT looking at apixaban versus warfarin for AF in haemodialysis, failed to recruit sufficient participants [106]. although the preliminary findings suggested no difference in terms of SSE and major bleeding.

The AXADIA study (Compare Apixaban and Vitamin K antagonists in patients with atrial fibrillation and End- stage kidney disease), is an open-label German clinical trial investigating apixaban 2.5 mg bd versus phenprocoumarin in patients on dialysis [107]. The primary outcome being safety in terms of risk of bleeding events and secondary outcome looking at efficacy in preventing thromboembolic events.

The fifth issue regards anticoagulant use in atrial fibrillation in patients with CrCl <50 ml/min. From the RCTs in people with AF and a CrCl 25–50 ml/min, there was no significant difference in stroke between the DOACs and warfarin, except for dabigatran 150 mg which had a reduced risk of stroke and systemic embolism, HR 0.56 (0.37–0.85) [27]. In terms of primary safety outcomes from the RCT sub-analyses apixaban and edoxaban were found to have a significantly reduced risk of major bleeding compared to warfarin [28, 29]. This reduction in major bleeding was not seen with dabigatran and rivaroxaban and this may in part relate to their increased risk of GI bleeding presented in the RE-LY and ROCKET- AF trials [108, 109]. However, when looking at intracranial haemorrhage within the CKD sub-analyses, both dabigatran and edoxaban reported a reduced risk of intracranial haemorrhage compared to warfarin [27, 28]. Intracranial haemorrhage was not presented in the CKD sub-analyses of apixaban but the overall ARISTOTLE trial found reduced likelihood compared to warfarin [110]. These findings would suggest that in terms of their safety profile, apixaban and edoxaban may be preferable for AF anticoagulation in CrCl 25–50 ml/min given their non-inferior efficacy to warfarin. It should be noted that due to dabigatran having the highest degree of renal excretion (around 85%), with no license in Europe for those with a CrCl < 30 ml/min [111] its use is likely to be limited in patients with CKD.

The sixth point regards anticoagulant use in atrial fibrillation in patients with a CrCl <30 ml/min not on dialysis.  There were fourteen retrospective cohort studies that included patients with AF and a CrCl < 30 ml/min. Two studies found that warfarin reduced the risk of stroke versus no anticoagulation, one study found no difference and a fourth study identified an increased risk of stroke with warfarin as compared to no anticoagulation. Three studies showed that DOACs reduced the risk of stroke compared to warfarin with seven studies finding no difference in stroke between DOACs and warfarin. Unfortunately, there was significant heterogeneity between studies in terms of DOAC dosing (if reported) and how the results were presented in relation to renal function, making it difficult to draw conclusions.

In terms of major bleeding in CrCl < 30 ml/min, the majority of studies found lower bleeding with DOACS versus warfarin with the remainder finding similar rates of bleeding. A recent study by Sy et al. found that for AF in patients with eGFR < 60 ml/min/1.73 m^2^ including dialysis, DOACs had a lower risk of major bleeding compared to warfarin [112] which would support the safety profile of DOACs compared to warfarin in this population.

**Fig. 7:**
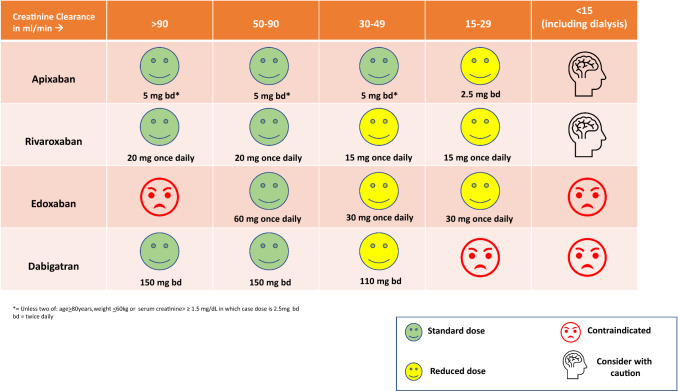
Authors suggested use of DOACs in patients with AF depending on renal function

In a population with a CrCl < 30 ml/min not on dialysis, anticoagulation should be considered on an individual patient basis with clinical assessment of the risks and benefits. From the limited available studies, DOACs may have a preferable safety profile over warfarin for patients with AF in this group. DOACs are not licensed in patients with a CrCl < 15 ml/min and more data is required before the safety and efficacy of DOACs in this group of patients are known. The authors of this review have presented their thoughts on the use of DOACs in patients with AF and varying degrees of renal impairment in Fig. 7, which are not dissimilar to a recent position statement published by the three Italian scientific societies [113].

One of the major limitations to this review are the small number of RCTs identified. Most studies are retrospective cohort studies with significant heterogeneity making performance of meta-analysis difficult. Differences existed in the definitions of major bleeding and stroke, renal function reporting (range and estimating equation), and anticoagulant doses used, making it difficult to draw conclusions from the data.

## Conclusions

In patients with VTE or AF and a CrCl ≥ 30 ml/min, DOACs may be preferable to warfarin with lower major bleeding and similar efficacy. Apixaban may be a safer and more effective option for the treatment of VTE in dialysis patients compared to warfarin, although this systematic review highlights the need for further studies in patients taking anticoagulation for VTE treatment or VTE prophylaxis in patients with advanced CKD (CrCl < 30 ml/min) to identify the safest and most effective options. The VERDICT trial is currently ongoing and may support future prescribing practice for VTE in more advanced CKD. For AF there is limited information for patients with a CrCl < 30 ml/min in terms of the risks and benefits of anticoagulation in reducing the risk of stroke. Until further data is available individualised decisions made with the patient and clinician are essential in this population, especially for those on dialysis. The ongoing AVKDIAL, AXADIA, DANWARD, SACK and SAFE-D trials in dialysis patients may help guide management of AF in the future.

## Supplementary Information

Below is the link to the electronic supplementary material.

Supplementary file1 (DOCX 16 kb)

Supplementary file2 (DOCX 33 kb)

Supplementary file3 (DOCX 12 kb)

Supplementary file4 (DOCX 14 kb)

## Data Availability

The data underlying this article are available in the article and in its online supplementary material.
