# Supplementary material for: A systematic review of the efficacy and safety of anticoagulants in advanced chronic kidney disease
Source: J Nephrol. 2022 Aug 25;35(8):2015–33. doi: 10.1007/s40620-022-01413-x (PMC9584987; doi:10.1007/s40620-022-01413-x)
Supplement: Supplementary file 1 — Supplementary file1 (DOCX 16 kb) [file 40620_2022_1413_MOESM1_ESM.docx]

| Reference | Bias from randomisation | Bias due to deviations from intended intervention | Bias due to missing outcome data | Bias in the measurement of the outcome | Risk of bias in selection of reported result | Overall Risk of bias judgement |
| --- | --- | --- | --- | --- | --- | --- |
| Goldhaber, 2017 | Low | Low | Low | Low | Low | Low |
| Agnelli, 2013 | Low | Low | Low | Low | Low | Low |
| Bauersachs, 2014 | Low | Some concerns | Low | Low | Low | Low |
| Buller, 2013 | Low | Low | Low | Low | Low | Low |
| De Vriese, 2020 | Low | Some concerns | Low | Low | Some concerns | Some concerns |
| Hijazi, 2013 | Low | Low/some concerns | Low | Low | Low | Low |
| Fox, 2011 | Low | Low | Low | Low | Low | Low |
| Hohnloser, 2012 | Low | Low | Low | Low | Low | Low |
| Bohula, 2016 | Low | Low | Low | Low | Low | Low |

Supplementary table 1. Risk of Bias assessment of included randomised controlled trials using the RoB 2 template

Supplementary table 2. Risk of Bias assessment of included cohort studies using the ROBINS-I tool

| Reference | Bias due to confounding  (Baseline and time-varying) | Bias in selection of participants | Bias in classification of interventions | Bias due to deviations from intended interventions | Bias due to missing data | Bias in measurement of outcomes | Bias in selection of the reported result | Overall bias |
| --- | --- | --- | --- | --- | --- | --- | --- | --- |
| Chan, 2013 | Moderate | Moderate | Moderate | Low | Low | Moderate | Low | Moderate |
| Green, 2017 | Serious | Serious | Moderate | No information | Moderate | Moderate | Low | Serious |
| Kooiman, 2013 | Moderate | Low | Moderate | No information | Low | Moderate | Low | Moderate |
| Siontis, 2018 | Low | Low | Low | No information | Low | Low | Low | Low |
| Di Lullo, 2018 | Serious | Moderate | Moderate/Serious | No information | Low | Moderate/Serious | Low | Serious |
| Chan, 2015 | Moderate | Low | Low | Moderate | Low | Low | Moderate | Moderate |
| Yoon, 2017 | Moderate | Low/Moderate | Low/Moderate | No information | Low/Moderate | Moderate | Low | Moderate |
| Chang, 2019 | Moderate | Low/Moderate | Low/Moderate | Moderate | Moderate | Moderate | Moderate | Moderate |
| Genovesi, 2017 | Moderate | Low/Moderate | Moderate | Low | Low/Moderate | Moderate | Low | Moderate |
| Olesen, 2012 | Moderate | Moderate | Low | Moderate | Low | Moderate | Low | Moderate |
| Yodogawa, 2015 | Moderate | Moderate | Moderate | Moderate | Low | Moderate | Moderate | Moderate |
| Shin, 2018 | Low/Moderate | Low | Moderate | No information | Low/Moderate | Low/Moderate | Low | Moderate |
| Laugesen, 2019 | Low | Low | Moderate/Serious | Low | Moderate | Moderate | Low | Moderate |
| Weir, 2020 | Low/Moderate | Low | Low | No information | Low | Moderate | Low | Moderate |
| Coleman, 2019 | Moderate | Low | Moderate | Low | Low | Moderate | Low | Moderate |
| Jun, 2017 | Moderate | Low | Low | No information | Moderate | Moderate | Low | Moderate |
| Wetmore, 2020 | Low | Low | Low | Low | No information | Low | Low | Low |
| Shen, 2015 | Low | Low | Low | No information | Moderate | Low | Low | Moderate |
| Kai, 2017 | Moderate | Low | Moderate | No information | Moderate | Moderate | Low | Moderate |
| Wakasugi, 2014 | Moderate | Moderate/Serious | Moderate | No information | Low | Moderate/Serious | Low | Serious |
| Chan, 2016 | Seriuos/Critical | Serious | Serious | No information | Low | Moderate | Low | Serious |
| Tan, 2017 | Moderate | Low | Low | No information | Low | Low | Low | Low |
| Wang, 2015 | Moderate | Moderate/Serious | Moderate | No information | Low | Moderate/Serious | Low | Serious |
| Lai, 2019 | Serious | Moderate | Moderate | No information | Moderate | Moderate | Low | Serious |
| Shah, 2014 | Low | Moderate | Low/moderate | Moderate | Moderate | Moderate | Low | Moderate |
| Phan, 2019 | Serious | Low | Moderate | No information | Low | Moderate | Low | Serious |
| Genovesi, 2015 | Low | Moderate | Moderate | Moderate | Moderate | Low | Low | Moderate |
| Mitsuma, 2015 | Serious | Moderate | Low | Moderate | Moderate | Moderate | Moderate | Serious |
| Chan, 2009 | Moderate | Low | Low | Low | Low | Moderate | Low | Moderate |
| Winkelmayer, 2011 | Moderate | Low | Low | No information | Low | Moderate | Moderate | Moderate |
| Garg, 2016 | Moderate | Moderate | Moderate/Serious | No information | Low/Moderate | Moderate | No information | Moderate/ Serious |
| Mavrakanas, 2020 | Low | Low | Low | Low | Low | Low | Low | Low |
| Lin, 2021 | Moderate | Low | Low | Low | Low | Low | Low | Low |
| Konigsbrugge, 2021 | Moderate | Low | Low | Low | Low | Low | Low | Low |
| Heleniak, 2020 | Moderate | Low | Low | Low | Low | Moderate | Low | Low/Moderate |
| Chantrarat, 2020 | Low | Moderate | Low | Low | Low | Low | Moderate | Low/Moderate |
| Agarwal, 2021 | Low | Low | Low | Low | Low | Low | Low | Low |
| See, 2021 | Low | Low | Low | Low | Low | Low | Low | Low |
| Wetmore, 2022 | Low | Low | Moderate | Low | Low | Low | Low | Low/moderate |
| Vaitsiakhovich, 2022 | Low | Low | Low | Low | Low | Low | Low | Low |
| Akbar, 2022 | Moderate/Serious | Moderate | Moderate | Moderate | Moderate | Serious | Moderate | Serious |
| Wetmore, 2022 AJKD | Low | Low | Low | Low | Low | Low | Low | Low |
| Sy, 2022 | Low | Low | Low | Low | Low | Low | Low | Low |
| Koretsune, 2022 | Low | Low | Low | Low | Low | Low | Low | Low |
