# Supplementary material for: A systematic review of the efficacy and safety of anticoagulants in advanced chronic kidney disease
Source: J Nephrol. 2022 Aug 25;35(8):2015–33. doi: 10.1007/s40620-022-01413-x (PMC9584987; doi:10.1007/s40620-022-01413-x)
Supplement: Supplementary file 2 — Supplementary file2 (DOCX 33 kb) [file 40620_2022_1413_MOESM2_ESM.docx]

| Supplementary Table 4. Study characteristics of included AF studies | | | | | | | | |
| --- | --- | --- | --- | --- | --- | --- | --- | --- |
| Study | Study design | Follow up | Treatment (n) | Comparator (n) | Renal function | Age, years | Risk stroke | Data collection |
| De Vriese, 2021 | RCT | 1.88 years (median) | Rivaroxaban 10mg od 46  + Riva with Vit K2 42 | Warfarin 44 | Haemodialysis | Median  Warfarin 80.3  Riva  79.9  Riva+Vit K2  79.6 | Warfarin 4.8  Riva  4.7  Riva+Vit K2  4.5 | Patient follow up |
| Fox, 2011 | RCT  Subanalysis ROCKET-AF | 707 days | Rivaroxaban 15mg od  (1474) | Warfarin 1476 | CrCl 30-49ml/min | Median  Riva79  Warf79 | 3.68  3.67 | Patient follow up |
| Hijazi, 2013 | RCT subanalysis RE-LY | 2 years (mean) | Dabigatran 150mg bd 1232  Dabigatran 110mg bd 1196 | Warfarin 1126 | CrCl30-<50ml/min using CKD-EPI | Mean  75.2 for CrCl<50 | CHADS2 for crcl<50  2 36.5%  >3 44.7% | Patient follow up |
| Hohnloser, 2012 | RCT subanalysis ARISTOTLE | 1.8 years | Apixaban 5mg bd 769  Apixaban 2.5mg bd 733 | Warfarin 1515 | CrCl<50ml/min  (25-49ml/min) | Mean  CrCl<50ml/min 77.6 | CHADS2  2.6 or  CHADS2- VASC 4.4 | Patient follow up |
| Bohula, 2016 | RCT  Subanalysis ENGAGE-AF TIMI 48 | 2.8 years | Edoxaban 30mg od 1334  Edoxaban 60mg od 1014 | Warfarin 1361 vs 30mg  1062 vs 60mg | CrCl 30-50ml/min | Median  CrCl 30-50ml/min  79 | CHADS2 3.1  CHADS2-VASC 5 | Patient follow up |
| Mavrakanas, 2020 | Retrospective cohort study  Propensity matched | 155 days | Apixaban 521  207 on 5mg bd  257 on 2.5mg bd | No treatment 1561 | Haemodialysis and peritoneal dialysis | Mean  Apixaban 68  No treatment 69 |  | US renal data system (USRDS) |
| Shen, 2015 | Retrospective cohort study | ITT was around 1.4 years | Warfarin 1838 | No treatment 10446 | Haemodialysis | Mean  Warfarin 61.8  No treatment 61.9 | CHADs2 >2  92% vs 90.9%  >3  70.9 vs 69.3% | USRDS |
| Kai, 2017 | Retrospective cohort  Propensity matched | 2.1 years | Warfarin 888 | No treatment 888 | Haemodialysis | Mean  Warfarin 68.9  None 67.3 | CHADS2 VASC 5.2  5.2 | Internal dialysis registry |
| Wakasugi, 2014 | Prospective cohort  Propensity matched | 110 person years | Warfarin 28 | No treatment 32 | Haemodialysis | Mean  Warfarin 67.8  None 68.4 | CHADs 2  >2 81% warfarin  90% none users | Dialysis |
| Chan, 2016 | Cohort study | 18 months | Warfarin 67 | No treatment 118 | Peritoneal dialysis | Mean  Warfarin 69.4 vs 69.5 | CHADS2 VASC  3.46 vs2.97 none | Hospitals information network |
| Wang, 2015 | Retrospective cohort | 4.4 years | Warfarin 59 | No treatment 82 | Dialysis | Mean  Warfarin 59.8 vs 62.1 | CHADS2 Vasc  Warf 3.9 vs 3.7 | Departmental database |
| Lai, 2009 | Retrospective cohort | Warfarin 31 months  None 23 | Warfarin 232 | No treatment 167 | eGFR<60 ml/min/1.73m2  MDRD  33% eGFR<15  23% haemodialysis | Mean  Warfarin 73  None 77 | n/a | Chart review |
| Tan, 2019 | Retrospective cohort | Until death, transplant or Medicare cessation | Warfarin 1651 | No treatment 4114 | Dialysis (Pd and HD) | Mean  Warfarin 74.4  None 74. | CHADS2VAsc score 4-8  Warf 83.5%  None 84.3% | USRDS |
| Shah, 2014 | Retrospective cohort | Until an event | Warfarin 756 | No treatment 870 | Dialysis | Mean 75 | CHADS2>2  Warfarin 77%  None 69% | Databases in Ontario + Quebec |
| Phan, 2019 | Retrospective cohort study | 2 years | Warfarin 115 | No treatment 361 | Dialysis | Mean  Warfarin 67.3  None 62.9  P=0.0001 | CHADS2-VASc >2  Warfarin 99.1%  None 95.3% | Internal dialysis registry |
| Genovesi, 2015 | Prospective cohort | 2 years or death | Warfarin 134 | No treatment 156 | Haemodialysis | Age >75years 50% in both groups | CHADS2-VASc 5-9  Warfarin 40.3%  None 48.1% | Dialysis centres |
| Mitsuma, 2015 | Retrospective cohort study | 3 years | Warfarin 27 | No treatment 55 | Haemodialysis | Mean 71.2 years | Not reported | Dialysis registry |
| Chan, 2009 | Retrospective cohort | Mean 1.6years | Warfarin 746 | No treatment 925 | Haemodialysis | Mean  72 | Warfarin 2.74  None 2.58 | Fresenius Medical care databases |
| Winkelmayer, 2011 | Retrospective cohort | Not specified | Warfarin 237 | No treatment 948 | Dialysis | Mean  Warfarin 68.6  None 70.1 | Not recorded | USRDS |
| Garg, 2016 | Retrospective cohort | Mean 2.1 years | Warfarin 119 | No treatment 183 | Haemodialysis | Mean  Warfarin 75  None 78 | CHADS2-VASc 5-9  Warfarin 47.1%  None 38.3% | Hospital database |
| Jun, 2017 | Retrospective cohort | 1 year | Warfarin 7446 | No treatment 7446 | eGFR 45-59 ml/min/1.73m2  30-44  <30  No dialysis patients | Mean  Warfarin 78.2  None 78.1 | CHADS2 4-6  Warfarin 11.8%  None 12.1% | Health ministry data |
| Yoon, 2017 | Retrospective cohort | 15.9 months | Warfarin 2774 | No treatment 2774 | Haemodialysis | Mean  67.6 both groups | CHADS2-VASc >3  Warfarin 44.7% None 44.6% | Korean claims database |
| Genovesi, 2017 | Retrospective cohort | 4 years or death | Warfarin 134 | No treatment 150 | Dialysis | Mean  76 both groups | CHADS2-VASc 5-9  Warfarin 42.9%  None 43.3% | Dialysis centre |
| Olesen, 2012 | Retrospective cohort | - | Warfarin 178 | No treatment 678 | Dialysis | Mean 66.8 | CHADS2-VASc>2  77% | Danish National registries |
| Yodogawa, 2015 | Retrospective cohort | Until an outcome | Warfarin 30 | No treatment 54 | Haemodialysis | Mean  Warfarin 69.5  None 70.4 | CHADS2  Warfarin 1.7  None 1.5 | Hospital records |
| Shin, 2018 | Retrospective cohort | - | DOACS(Apixaban and dabigatran)  1120  21% of DOAC users were prescribed a reduced dose | Warfarin 1120 | eGFR30-59 ml/min/1.73m2  eGFR<30 4%  No dialysis | Mean  DOACs 73  Warfarin 72 | CHADS2-VASc 4 in both groups | Hospital records |
| Weir, 2020 | Retrospective cohort | Upto 2 years | Rivaroxaban 781  60% 15mg od  15% 20mg od  25%<15mg od | Warfarin 781 | CrCl15-30ml/min 81.3%  CrCl<15 non dialysis 3.7%  Dialysis 15% | Mean  Both 79.9 | CHADS2-VASc 4.5 | Optum health database |
| Coleman, 2019 | Retrospective cohort | Until outcome or discontinuation anticoagulant | Rivaroxaban 1896  61.3% on 20mg od , remainder on 15mg od | Warfarin 4848 | eGFR 15-29 ml/min/1.73m2 15%  eGFR<15 85% | Mean  72 both | CHADS2-VAsc 4 in both | Database |
| Laugesen, 2019 | Retrospective cohort | 1 year | DOAC (apixaban or dabigatran) 552  No dosing information | Warfarin 1008 | 95% had eGFR<59 ml/min/1.73m2 | Median  DOAC 80  Warfarin 78 | Not recorded | Danish nationwide database |
| Wetmore, 2020 | Retrospective cohort | - | Apixaban 6738  Rivaroxaban 3904  Dabigatran 1568  No dose information | Warfarin 10529 | eGFR<60 ml/min/1.73m2 | Mean 78 | CHADS2-VASc 5.3 | Database |
| Chan, 2015 | Retrospective cohort | Up to 2 years | Rivaroxaban 244 (32.1% 20mg daily, 67.8% 15mg daily)  Dabigatran 281 (15.3% 150mg bd, 84.7% 75mg bd) | Warfarin 8064 | Haemodialysis | Mean  Rivaroxaban 66.9  Dabigatran 68.4  Warfarin 70.6 | CHADS2  Rivaroxaban 2.2  Dabigatran 2.3  Warfarin 2.4 | Fresenius medical care database |
| Di Lullo, 2018 | Retrospective cohort | 16 months | Rivaroxaban 15mg daily 247 | Warfarin 100 | eGFR 15-45 ml/min/1.73m2 | Mean  66 in both | Not recorded | Outpatient charts |
| Siontis, 2018 | Retrospective cohort | Upto 5 years, death or anticoagulant switch | Apixaban 2351  5mg bd 1034  2.5mg bd 1317 | Warfarin 23172 | Dialysis | Mean  68 in both groups | CHADS2-Vasc  Apixaban | USRDS |
| Chang, 2019 | Retrospective cohort | Upto 5 years or until outcome | DOAC 280  Dabigatran, Rivaroxaban, Edoxaban, Apixaban at varying doses  Warfarin 520 | No treatment 2971 | eGFR 15-29 ml/min/1.73m2  eGFR<15  (25% of patients on dialysis) | Median  None 78  DOAC 79  Warfarin 76 | CHADs2-VASc  None 4.5  DOAC 4.7  Warfarin 4.6 | Hospital database |
| Chantrarat, 2020 | Prospective cohort | 25.5+10.6 months | Warfarin 1138  DOACs 121 | No treatment 335 | CKD stage 3  CKD stage 4-5 | Mean  CKD stage 3 70  CKD stage 4-5 72.1 | CHA2DS2-VASc >2  CKD stage 3 n=1138  CKD stage 4&5 n=222 | 27 hospitals across Thailand |
| Agarwal, 2020 | Retrospective cohort | Upto 7.5 years or until outcome or death | Warfarin 6682 | No treatment 16089 | Haemodialysis | Mean  None 74.3  Warfarin 71.4 | CHA2DS2-VASc  None 6  Warfarin 5.1 | USRDS |
| Heleniak, 2020 | Prospective cohort | 26.3months | DOAC n=90  (Apixaban n=61  Rivaroxaban n=29) | Warfarin n=92 | eGFR 15-29ml/min/1.73m2 | Mean  DOAC 70.4  Warfarin 68.79 | CHA2DS2-VASc  DOAC 3.13  Warfarin 3.02 | Single centre |
| Konigsbrugge, 2021 | Prospective cohort | 870days | Phenprocoumon n= 61 | No treatment n=139 | Haemodialysis | Median  None 73  Phenprocoumon 70 | CHA2DS2-VASc  None 4  Phenprocoumon 3 | Multi-centre, Vienna |
| Lin, 2021 | Retrospective cohort | Upto 4 years or outcome | Rivaroxaban 173  88 10mg  67 15mg  18 20mg | Warfarin 3185 | eGFR<15ml/min/1.73m^2^ including dialysis | Mean  69 in both | CHA2DS2-VASc >4  Rivaroxaban 56%  Warfarin 53% | Taiwan national research database |
| See, 2021 | Retrospective cohort | Upto 5.5 years or until study outcome | Warfarin 448  DOACs 488 | No treatment 2977 | Dialysis | Mean  DOACs 74.3  Warfarin 75.2  None 71.1 | CHA2DS2-VASc  DOACs 4.5  Warfarin 4.7  None 4.1 | Taiwan national research database |
| Vaitsiakhovich, 2022 | Retrospective cohort | Warfarin median 115 days  Rivaroxaban median 119 days | Warfarin 5903 | Rivaroxaban 15mg daily 1465 | eGFR 15-60ml/min/m^2^ | Median  Warfarin78 Rivaroxaban 79 | CHA2DS2-VASc  Warfarin 4.44  Rivaroxaban 4.41 | US IBM Watson MarketScan databases |
| Akbar, 2022 | Retrospective cohort | 11 months | Warfarin 44 | None 44 | Haemodialysis | Mean  Warfarin 51.2  None 53.2 | CHA2DS2-VASc≥2  Warfarin 23.8%  None 6.81% | Single-centre |
| Wetmore, 2022 | Retrospective cohort | Mean 567 days | Warfarin 12,517 | Apixaban as per label 2382  Apixaban dose below label (2.5mg bd) 2257 | Haemodialysis | Age (%) Warf, Apix label, Apix non-label  18-44 2.7, 3.7, 2.0  45-64 34.7, 39.0, 30.0  65-74 44.3, 43.0, 44.4  75-79 18.3, 14.3, 23.6 | CHA2DS2-VASc, mean  Warfarin 4.5 Apixaban label 4.3  Apixaban non-label 4.7 | Medicare database |
| Sy, 2022 | Retrospective cohort | Not stated | Warfarin 5960 (propensity score matched) | No anticoagulation 5960 (propensity score matched) | Transitioning to dialysis | Mean 74 | CHA2DS2-VASc score, median 6 | USRDS |
| Koretsune, 2022 | Retrospective cohort | Median follow up days  Apixaban 717  Warfarin 735 | Apixaban 1394  17.9% 5mg bd  80.4% 2.5mg bd  1.6% other dose | Warfarin 1394 | CrCl15-49ml/min | Mean  Apixaban 81.8　 Warfarin81.5 | CHA2DS2-VASc  Apixaban 4.0  Warfarin 4.0 | 402 Japanese institutions |

RCT randomised controlled trial, USRDS United States renal data system, CHA2DS2-VASc (Congestive Cardiac Failure, Hypertension, Age>75 2 points, Diabetes, Stroke 2 points, Vascular disease, Age 64-75 1 point, Sex female), CHADS2 (Congestive heart failure, Hypertension, Age > 75, Diabetes, previous Stroke/transient ischaemic attack (2 points)).

Supplementary table 5. Stroke outcomes in studies relating to Atrial Fibrillation in CrCl<50ml/min

| Study | Renal function | Definition of stroke | Treatment Incidence | Comparator Incidence | HR 95% CI |
| --- | --- | --- | --- | --- | --- |
| De Vriese, 2021 | Haemodialysis | Ischaemic, haemorrhagic or uncertain type. Systemic embolism was acute vascular occlusion of limb or organ identified via imaging, surgery or autopsy, | Pooled rivaroxaban outcomes  6 | 9 | n/a  p=ns |
| Fox, 2011 | CrCl 30-49ml/min | Composite of all stroke and SE | 2.32 per 100person years | 2.77 | 0.84(0.57-1.23) |
| Hijazi, 2013 | CrCl <50ml/min | Fatal or non-fatal stroke and systemic embolism | Dabigatran 150  36  Dabigatran 110  52 | Warfarin  57 | Dabigatran 150 vs warfarin  0.56 (0.37-0.85)  Dabigatran 110 vs warfarin  0.85 (0.59-1.24) |
| Hohnloser, 2012 | Crcl<50ml/min | SSE | 54 | 69 | 0.79 (0.55-1.14) |
| Bohula, 2016 | CrCl 30-50ml/min | SSE | Edoxaban 60mg 82  Edoxaban 30mg 110 | Warf 91  Warf 91 | 0.87 (0.65-1.18)  1.22 |
| Mavrakanas, 2020 | Dialysis | Stroke, TIA or systemic embolism | Overall Apixaban 7.5 per 100person years  Apixaban 5mg bd 13.6 per 100 patient years  Apixaban 2.5mg bd 5.7 per 100 patient years | Overall no treatment 7 per 100 person years  7.6 per 100 patient years  6.1 per 100 patient years | 1.24 (0.69-2.23)  aHR 2.24 (1.03-4.86) p=0.04  aHR 1.11 (0.43-2.85) p=ns |
| Shen, 2015 | Haemodialysis | Ischaemic stroke  Haemorrhagic stroke | 63 (2.3 per 100pt years)  29 (1 per 100 pt years) | 503 (3.4per 100pt year)  192 (1.3 per 100pt years) | 0.68 (0.47-0.99)  0.82 (0.37-1.81) |
| Kai, 2017 | Haemodialysis | Ischaemic stroke  Haemorrhagic stroke | 58 (2.8 per 100pt years)  20 (0.9 per 100 pt years) | 88 (4.8 per 100pt years)  12 (0.6 per 100 pt years) | RR 0.6 (0.4-0.8)  RR 1.5 (0.7-3.4) |
| Wakasugi, 2014 | Haemodialysis | Ischaemic stroke | 8 (14.8 per 100pt years) | 5 (8.9 per 100pt years) | HR 1.94 (0.63-5.93) |
| Chan, 2016 | Peritoneal dialysis | Ischaemic stroke | 0 | 11 | HR 0.19 (0.06-0.65) p=0.01 |
| Wang, 2015 | Dialysis | Iscahemic stroke and other arterial embolism | 8 | 11 | n/a |
| Lai, 2009 | eGFR<60 ml/min/1.73m2 | Thromboembolic stroke | 21 | 43 | P<0.001 |
| Tan, 2019 | Dialysis | Any stroke ( ischaemic stroke, cerebral thrombosis cerebral ischaemia and other cerebrovascular disease) | 124 | 826 | HR 0.92 (95%CI 0.75-1.12) |
| Shah, 2014 | Dialysis | Ischaemic cerebrovascular disease including TIA and retinal infarct | 52 | 55 | HR 1.17 (95%CI 0.79-1.75) |
| Phan, 2019 | Dialysis | Ischaemic stroke | 10 | 11 | RR 2.6 (95%1.1-6.2) |
| Genovesi, 2015 | Dialysis | Thromboembolic events | 8  3.7 per 100 patient years | 9  3.7 per 100 patient years | NR |
| Mitsuma, 2015 | Dialysis | Ischaemic stroke and Systemic embolism | 3 | 5 | NR |
| Chan, 2009 | Dialysis | Hospitalisation from Ischaemic stroke or TIA | 65 | 40 | HR=1.89 (95% CI 1.16-3.09) |
| Winkelmayer, 2011 | Dialysis | Ischaemic stroke | 29 | 135 | HR 1.03 (0.62-1.7) |
| Garg, 2016 | Dialysis | Ischaemic stroke | 13 | 21 | aHR 0.93 (95%CI 0.49-1.82) |
| Jun. 2017 | eGFR 45-59 ml/min/1.73m2  eGFR 30-44  eGFR <30 | Stroke and TIA |  | - | aHR 0.6 (0.44-0.84)  aHR 0.59 (0.38-0.94)  aHR 0.54 (0.26-1.13) |
| Yoon, 2017 | Dialysis | Ischaemic stroke | 204 | 201 | P=0.877 |
| Genovesi, 2017 | Dialysis | Thromboembolic events | 25 | - | HR0.44 (0.16-1.2) |
| Olesen, 2012 | Dialysis | Stroke and systemic embolism | - | - | HR 0.44 (0.26-0.74) p=0.002 |
| Yodogawa, 2015 | Dialysis | Stroke (haemorrhagic and ischaemic) | 2 | 5 | P=0.87 |
| Shin, 2018 | eGFR<60 ml/min/1.73m2 | Ischaemic stroke | 86 | 92 | HR 1.02 (0.76-1.37) |
| Weir, 2020 | CrCl<30 | Stroke and systemic embolism requiring hospitalisation | 1.8 per 100 patient years | 1.96 per 100 patient years | HR 0.93 (0.46-1.9) p=0.85 |
| Coleman, 2019 | eGFR<30 ml/min/1.73m2 | Stroke and systemic embolism | 1.10 per 100 patient years | 2.16 per 100 patient years | HR 0.55 (0.27-1.1) |
| Laugesen, 2019 | eGFR<59 ml/min/1.73m2 | Stroke and systemic embolism | 11 | 21 | HR 0.83(0.39-1.78) |
| Wetmore, 2020 | eGFR<60 ml/min/1.73m2 | Stroke and systemic embolism | Apixaban 1.74 per 100pt years  Dabigatran 2.25 per 100 patient years  Rivaroxaban 1.84 per 100 patient years  ITT analysis | Warfarin 1.9 per 100 patient years  ITT analysis | Apixaban HR 0.83(0.68-1.02)  Rivaroxaban HR 0.94(0.73-1.02)  Dabigatran HR 1.19(0.92-1.54) |
| Chan, 2015 | Dialysis | Embolic stroke or arterial embolism | Rivaroxaban 8  Dabigatran 13 | Warfarin 244 | Dabigatran Rate ratio 1.71 (0.97-2.99)  Rivaroxaban  Rate ratio 1.8 (0.89-3.64) |
| Di Lullo, 2018 | eGFR15-45 ml/min/1.73m2 | Stroke and systemic embolism | 0 | 25 | - |
| Siontis, 2018 | Dialysis | Stroke and systemic embolism | 81 | 373 | HR 0.88 (0.69-1.12) p=0.29 |
| Chang, 2019 | eGFR15-29 ml/min/1.73m2  eGFR<15 | Ischaemic stroke and systemic embolism | eGFR15-29  DOAC 3  Warfarin 22  eGFR<15  DOAC 0  Warfarin 20 | eGFR15-29  39  eGFR<15  38 | aHR 1.3(0.4-4.2)  aHR 2.7(1.5-5.0)  n/a  aHR3.7(2-6) |
| Chantrarat, 2020 | CKD stage 3-5 | Ischaemic stroke/TIA | NOACs 0  Warfarin 45 | 14 | OAC versus none p=0.602  Warfarin vs NOAC p=0.026  Warfarin versus none p=0.854  NOAC versus none p=0.026 |
| Agarwal, 2020 | Haemodialysis | Ischaemic stroke/TIA | 105.5 per 1000 patient years | 107.3 per 1000 patient years | Adjusted HR 1.23 (1.15-1.32) |
| Heleniak | eGFR 15-29ml/min/1.73m2 | Stroke/TIA/systemic Embolism | NOAC n=11 | Warfarin n=7 | P=0.3 |
| Konigsbrugge, 2021 | Haemodialysis | Ischaemic stroke/TIA/Systemic embolism | - | - | Subdistribution HR  1.41 (1.09-4.07) |
| Lin, 2021 | eGFR<15ml/min/1.73m2 | Ischaemic stroke/systemic embolism | n=10 | n=520 | Adjusted SHR 0.36 (0.17-0.79) p=0.01 |
| See, 2021 | Dialysis | Ischaemic stroke/systemic embolism | Overall anticoagulant 5.84 per 100 pt years  DOAC 6.67 per 100 pt years Warfarin 5.3 per 100 pt years | None 3.62 per 100 pt years | DOAC vs Warfarin  HR 1.21 0.76-1.92) p=0.4183  Anticoagulation vs None HR 1.54 (1.29-1.84) p=<0.001 |
| Vaitsiakhovich, 2022 | eGFR 15-60ml/min/m2 | Ischaemic stroke | 1.06 per 100 pt years | 0.77 per 100 pt years | HR 0.77 (0.33-1.82) p=0.55 |
| Akbar, 2022 | Haemodialysis | Ischaemic stroke | 34.09% | 18.18% | P=0.003 |
| Wetmore, 2022 | Haemodialysis | Ischaemic stroke and Systemic Embolism | 2.1 per 100 pt years | Apixaban label 2 per 100 pt years  Apixaban non-label 1.9 per 100 pt years | HR (95% CI)  Label 0.89 (0.65, 1.21)  Non-label 0.85 (0.62, 1.17) |
| Sy, 2022 | Transitioning to dialysis | Ischaemic stroke | 3.10 per 100 pt-years | 2.99  per 100 pt-years | HR 1.04,  95% CI: 0.89–1.21, p = 0.66 |
| Koretsune, 2022 | CrCl 15-49ml/min | SSE | 1.62 per 100 pt years | 2.7 per 100 pt years | HR (95%CI)  0.60  (0.40–0.90)  P =0.01 |

ITT Intention to treat, SE systemic embolism, TIA transient ischaemic attack, HR hazard ratio, aHR adjusted hazard ratio, RR relative risk, DOAC direct oral anticoagulant, OAC oral anticoagulation, n/a not applicable, CHA2DS2-VASc (Congestive Cardiac Failure, Hypertension, Age>75 2 points, Diabetes, Stroke 2 points, Vascular disease, Age 64-75 1 point, Sex female), HASBLED (Hypertension, Abnormal renal/liver function, Stroke, Bleeding tendency, Labile INR, Age>65)

Supplementary table 6. Major bleeding outcomes in AF studies with patients with CrCl<50ml/min

| Study | Renal function | Definition of bleeding | HASBLED | Treatment incidence | Comparator incidence | HR |
| --- | --- | --- | --- | --- | --- | --- |
| De Vriese, 2021 | Haemodialysis | Life threatening is fatal, symptomatic intracranial, decrease in Hb of 5g/dL requires a 4unit transfusion or surgery. Major bleeding transfusion of 2 units or Hb drop 2g/dL or more. | Rivaroxaban 4.6  Rivaroxaban 4.7 | Life threatening 11  Major 12 | 12  Major 18 | n/a  p=0.033  p=0.015 |
| Fox, 2011 | CrCl 30-49ml/min | Composite of major and CRNMB  Intracranial haemorrhage | n/a | 17.82 per 100 patient years  0.71 | 18.28  0.88 | 0.98 (0.84-1.14)  0.81 (0.41– 1.60) |
| Hijazi, 2013 | CrCl<50ml/min | Major bleeding with Hb drop >2g/dL or >2 units transfusion or bleeding into a critical area/organ as per ISTH  Intracranial haemorrhage | n/a | Dabigatran 150  129  Dabigatran 110  122  Dabigatran 150  9  Dabigatran 110  11 | Warfarin 116  Warfarin 26 | Dabigatran 150 vs warfarin  1.01 (0.79-1.3)  Dabigatran 110 vs warfarin  0.99 (0.77-1.28)  Dabigatran 150 vs warfarin  0.31 (0.14–0.66)  Dabigatran 110 vs warfarin  0.40 (0.20–0.80) |
| Hohnloser, 2012 | CrCl <50ml/min | Major bleeding with Hb drop >2g/dL or >2 units transfusion or bleeding into a critical area/organ as per ISTH | Apixaban 2.3  Warfarin 2.3 | 73 | 142 | 0.5 (0.38-0.66) |
| Bohula, 2016 | CrCl 30-50ml/min | Major bleeding as per ISTH  Intracranial haemorrhage | CrCl 30-50ml/min | Edoxaban 60mg 100  Edoxaban 30mg 50  Edoxaban 60mg 17 | Warf 132  Warf 132  Warf 36 | 0.76 (0.58-0.98)  0.37  0.46 (0.26–0.82) P=0.009 |
| Mavrakanas, 2020 | Dialysis | Major bleeding: bleeding resulting in death or intracranial bleed  Clinically important bleeding:  Bleeding resulting in death. At a critical site (intraocular, intracranial, retroperitoneal, airway, pericardial, intra-articular) or any bleeding of gastrointestinal, urinary tract or gynaecological that led to hospitalisation | n/a taken into account in when adjusting HR | **Major bleeding** Overall apixaban 4.9 per 100person years  Apixaban 5mg bd 9.8 per 100 patient years  Apixaban 2.5mg bd 2.9 per 100 patient years  **Clinically important**  59.2 per 100 person years  Apixaban 5mg bd 77.2  Apixaban 2.5mg bd  51.4 | **Major bleeding**  Overall no treatment 1.6 per 100 person years  1.7 per 100 patient years  1.4 per 100 patient years  56.9 per 100 person years  57.2  54.7 | aHR 2.76 (1.38-5.52) p=0.004  aHR 4.61 (1.91-11.15) p=0.001  aHR 2.02 (0.58-7.04) p=ns  aHR 1.15 (0.9-1.46)  aHR 1.36 (0.94-1.96)  aHR 1.03 (0.71-1.47) |
| Shen, 2015 | Haemodialysis | Gastrointestinal bleeding | >3  70.9% warfarin users  69.3% none | 153 (5.9 per 100pt years) | 833 (5.9 per 100 pt years) | 1 (0.69-1.44) |
| Kai, 2017 | Haemodialysis | Gastrointestinal bleeding  Any bleed, defined by ICD9-CM | Warfarin 5  None 4.4 | Warfarin 111 (5.3 per 100 pt years)  Any bleed 181 (8.6 per 100 pt years) | 101 (5.5 per 100 pt years)  Any bleed 149 (8 per 100 pt years) | Rate ratio 1 (0.8-1.3)  RR 1.1 (0.9-1.4) |
| Wakasugi, 2014 | Haemodialysis | Major bleeding: Fatal bleeding or bleeding requiring hospitalisation | n/a | Warfarin 3 (5.3 per 100 pt years) | 4 (6.6 per 100 pt years) | HR 0.85 (0.19-3.64) |
| Chan, 2016 | Peritoneal dialysis | Intracranial haemorrhage | Warfarin 2.56  None 1.94 | 0 | 0 | No analysis |
| Wang, 2015 | Dialysis | Bleeding events included intracranial, gastrointestinal, dialysis site or other bleed | Warfarin 3.3  None 3.5 | Intracranial 4  Gastrointestinal 11 | Intracranial 0  Gastrointestinal 16 | P=0.029  P=1 |
| Lai, 2009 | eGFR<60ml/min/1.73m2  MDRD | Not formally defined but major bleeding reported includes GI bleeding, Intracerebral bleeding, transfusion for hemoptysis, retroperitoneal haematoma, intraperitoneal bleeding, pelvic haemorrhage, | Not reported | Overall major 32  Intracerebral 11 | Overall major 15  Intracerebral 3 | P=ns |
| Tan, 2019 | Dialysis | Major bleeding includes subarachnoid bleeding, intracerebral bleeding, GI bleeding, haematuria and other | High risk >3  Warfarin 49%  None 50.7% | 407 | 1559 | HR 1.5 (95% 1.33-1.68) |
| Shah, 2014 | Dialysis | Intracerebral bleeding, GI bleeding, intraocular bleeding, haematuria and unspecified location of bleeding | High risk >3  Warfarin 84%  None 86% | 149 | 126 | HR 1.41(95%1.09-1.81) |
| Phan, 2019 | Dialysis | Haemorrhagic stroke  GI bleed | Warfarin 4.6  None 4 | 2  7 | 3  22 | RR1.9 (0.3-11.6)  RR 0.9 (95% CI 0.4-2.2) |
| Genevosi, 2015 | Dialysis | Haemorrhagic events | HASBLED>3  Warfarin 59%  None 71.8% | 381  7.6 per 100 patient years | 29  11.8 per 100 patient years | NR |
| Mitsuma, 2015 | Dialysis | Major bleeding (haemorrhagic stroke, GI bleeding and others) | NR | 7 | 9 | NR |
| Chan, 2009 | Dialysis | Hospitalisation from bleeding | NR | 97 | 107 | HR 1.04 (95%CI 0.73-1.46) |
| Winkelmayer, 2011 | Dialysis | Haemorrhagic stroke  GI bleeding | NR | 11  48 | 21  216 | HR 2.38 (95%1.15-4.96)  HR 0.96 (0.7-1.31) |
| Garg, 2016 | Dialysis | Major bleeding is bleeding from any site requiring hospitalisation or blood transfusion | HASBLED 4-9  Warfarin 65.5%  None 59.5% | 26 | 26 | aHR 1.53 (0.94-2.51) |
| Jun, 2017 | eGFR45-59 ml/min/1.73m2  eGFR30-44  eGFR <30 | Hospitalisation for major bleeding to include intracranial, GI or other bleeding | HASBLED 3-6  Warfarin 3.61%  None 4.31% | - | - | aHR 1 (0.79-1.27)  aHR 0.82(0.62-1.09)  aHR 0.54(0.26-1.13) |
| Yoon, 2017 | Dialysis | Haemorrhagic stroke | HASBLED>2  Warfarin 78.6%  None 73.7% | 84 | 50 | P=0.003 |
| Genovesi, 2017 | Dialysis | Bleeding event | HASBLED 4-9  Warfarin 53.1%  None 53.3% | 55 | - | HR 1.16(0.48-2.82)  P=0.7 |
| Olesen, 2012 | Dialysis | Bleeding including intracranial, GI, urinary tract, airway, bleeding | HASBLED >3  22% | - | - | HR 1.27 (0.91-1.77) p=0.15 |
| Yodogawa, 2016 | Dialysis | Hospitalisation from bleeding | Not recorded | 3 | 1 | P=0.08 |
| Shin, 2018 | eGFR<60 ml/min/1.73m2 | Major bleeding including bleeding at a critical site or requiring a blood transfusion | 2 for both groups | 37 | 50 | HR 1.23(1.02-1.48) |
| Weir, 2020 | CrCl<30ml/min | GI, Intracranial or other major bleeding | 3.5 for both groups | 8.48 per 100 patient years | 9.39 per 100 patient years | HR 0.91 (0.65-1.28) p=0.6 |
| Coleman, 2019 | eGFR<30 ml/min/1.73m2 | Major bleeding requiring hospitalisation (Cunningham algorithm)  Intracranial haemorrhage | Not recorded | 3.73 per 100 patient years | 6.16 per 100 patient years | HR 0.68(0.47-0.99)  HR 0.19(0.02-1.56) |
| Laugesen, 2019 | eGFR<59ml/min/1.73m2 | Major bleeding requiring hospitalisation including GI, urogenital, airway, intraocular, intracranial | Not recorded | 15 | 155 | HR 0.47(0.26-0.84) |
| Wetmore, 2020 | eGFR<60ml/min/1.73m2 | Major bleeding as per Cunningham bleeding algorithm | 3.3 | Apixaban 2.3 per 100 patient years  Dabigatran 2.7 per 100 patient years  Rivaroxaban 3.23 per 100 patient years | 3.4-3.5 per 100 patient years  ITT analysis | HR apixaban 0.55(0.46-0.66)  HR rivaroxaban  0.86(0.73-1.02)  HR dabigatran 0.81(0.65-1.01) |
| Chan, 2015 | Dialysis | Major bleeding defined as bleeding that resulted in hospitalisation and death and including haemorrhagic stroke | HASBLED  Dabigatran 1.9  Rivaroxaban 1.8  Warfarin 1.9 | Dabigatran 83.1 per 100 patient years  Rivaroxaban 68.4 per 100 patient years | Warfarin 47.1 per 100 patient years | Dabigatran HR 1.76 (1.44-2.15)  Rivaroxaban HR 1.45 (1.09-1.93) |
| Di Lullo, 2015 | eGFR 15-45 ml/min/1.73m2 | Major bleeding that required hospitalisation | Not recorded | 2 | 8 | - |
| Siontis, 2018 | Dialysis | Major bleeding includes critical site bleeding, requirement of a transfusion or death | Not recorded | 129 | 715 | HR 0.72 (0.59-0.87) p<0.001 |
| Chang, 2019 | eGFR<30ml/min/1.73m2 | Major bleeding leading to hospitalisation | None 4  DOAC 3.7  Warfarin 4 | eGFR15-29 DOAC 14  Warfarin 25  eGFR<15  DOAC 4  Warfarin 38 | eGFR15-29  67  eGFR<15  61 | aHR 2.9(1.6-5.4)  aHR 1.9(1.2-3.1)  aHR 3.2 (1.1-9.3)  aHR 4.3 (2.8-6.5) |
| Chantrarat, 2021 | CKD stage 3-5 | Major bleeding as per ISTH | HASBLED >3  CKD stage 3  CKD stage 4&5 | DOAC n=4  Warfarin n=75 | n=11 | OAC versus none p=0.035  Warfarin versus DOAC p=0.157  Warfarin versus none p=0.023  NOAC versus none p=0.991 |
| Agarwal, 2020 | Haemodialysis | Haemorrhagic stroke, intracranial bleed or GI bleed | Not recorded | 144.7 per 1000 patient years | 128.9 per 1000 patient years | Adjusted HR1.34 (1.25,1.43) |
| Heleniak, 2020 | eGFR 15-29ml/min/1.73m2 | Major bleeding as per ISTH | Not recorded | NOAC n=4 | Warfarin n=9 | P=0.16 |
| Konigsbrugge, 2021 | Haemodialysis | Major bleeding as per ISTH | None 4  Phenprocoumon 3 | - | - | Adjusted subdistribution HR 2.28 (1.09-4.79) |
| Lin, 2021 | eGFR<15ml/min/1.73m2 | Major bleeding as per ISTH | ORBIT score >4 | n=23 | n=560 | Adjusted SHR 0.86 (0.5-1.47) p=0.59 |
| See, 2021 | Dialysis | All major bleedings that require hospitalisation, including all intracranial haemorrhage and GI bleeds | DOACs 3.7  Warfarin 3.6  None 3.6 | None 4.97 per 100 patient years | Overall anticoagulant 5.95 per 100 pt years  DOAC 7.07 per 100pt years  Warfarin 7.15 per 100 pt years | DOAC Vs Warfarin HR 0.98 (0.64-1.51) p=0.9373  Anticoagulation vs none HR 1.14 (0.97-1.34) p=0.122 |
| Vaitsiakhovich, 2022 | eGFR15-60ml/min/1.73m^2^ | Bleeding-related hospitalization (Cunningham) | Modified HAS-BLED (excludes INR)  Warfarin 3  Rivaroxaban 3 | 5.09 per 100 pt years | 5.90 per 100 pt years | HR 1.14 (0.83-1.58) P=0.42 |
| Akbar, 2022 | Haemodialysis | Haemorrhage events to include  Haemorrhagic stroke  Gastrointestinal bleeding  Minor bleeding | Unclear | 3.41% | 3.41% | P=1 |
| Wetmore, 2022 | Haemodialysis | Major bleeding to include bleeding events that were  (i) fatal, and/or (ii) involved a critical site, and/or (iii) required a blood transfusion | HAS-BLED, mean (SD)  3.0 (0.8)  2.9 (0.8)  3.1 (0.8) | 6.3 per 100 pt years | Label 4.5 per 100 pt years  Non-label 4.7 per 100 pt years | Label 0.67 (0.55, 0.81)  Non-label 0.68 (0.55, 0.84) |
| Sy, 2022 | Haemodialysis | Bleeding | HAS-BLED median 3 both groups (propensity matched group) | 8.27 per 100 pt-years | 8.31  per 100 pt-years | HR 1.03, 95% CI:  0.93–1.14, p = 0.57 |
| Koretsune, 2022 | CrCl 15-49ml/min | Major bleeding defined as per ISTH | HAS-BLED  Apixaban 3  Warfarin 3 | 1.84 per 100 pt years | 3.1 per 100 pt years | HR (95% CI)  0.59 (0.41–0.87)  P=<0.01 |

CRNMB Clinically relevant non-major bleed, ISTH International society thrombosis and haemostasis, Hb haemoglobin, HR hazard ratio, aHR adjusted hazard ratio, DOAC direct oral anticoagulant, OAC oral anticoagulation, GI gastrointestinal, MDRD modified diet in renal disease, n/a not applicable, HASBLED (Hypertension, Abnormal renal/liver function, Stroke, Bleeding tendency, Labile INR, Age>65)
