# Supplementary material for: A systematic review of the efficacy and safety of anticoagulants in advanced chronic kidney disease
Source: J Nephrol. 2022 Aug 25;35(8):2015–33. doi: 10.1007/s40620-022-01413-x (PMC9584987; doi:10.1007/s40620-022-01413-x)
Supplement: Supplementary file 3 — Supplementary file3 (DOCX 12 kb) [file 40620_2022_1413_MOESM3_ESM.docx]

Supplementary Table 3. Characteristics and outcomes of included VTE prophylaxis studies

| Study | Study design | Data collection | Follow up | Renal function | Treatment (n) | Comparator (n) | Age, years | Risk factors | Outcome measures | VTE incidence treatment vs comparator | RR VTE | Incidence treatment vs comparator | RR bleeding |
| --- | --- | --- | --- | --- | --- | --- | --- | --- | --- | --- | --- | --- | --- |
| Green 2017 (25) | Retrospective cohort study  Single centre | Inpatient | Minimum 2 days consecutive treatment | Haemodialysis | Enoxaparin 30mg SC od  n=75 | UFH 5000units SC tds  n=150 | Mean (SD)  Enoxaparin 67.2 +13.8  UFH  67.8+14.7 | Padua Score  Enoxaparin 3.5+1.4  UFH 4.2+1.3  Immobility  78.7% Enoxaparin  96%UFH  No cancer or thrombophilia patients were in either group | Confirmed DVT or PE during hospitalisation  Major bleeding  CRNMB both defined by ISTH  Any bleed | 0 vs 0 | Not applicable | Major 0 vs 0  CRNMB 0 vs 0  Any bleed 1 vs 1 | Not applicable |
| Chan 2013 (24) | Fresenius Medical Care North America Research Database | Dialysis patient follow up | Enoxaparin 135days  UFH 145days | Haemodialysis | Enoxaparin SC 20mg, 30mg, 40mg and 60mg od  n=2991 | UFH 5000units SC bd-tds  n=4730 | Age>60  Enoxaparin 67.1%  UFH 68% | Obesity (>30kg/m2)  Enoxaparin 28%  UFH 30%  Immobility  Enoxaparin 5.1%  UFH 9.8%  Previous VTE  Enoxaparin 8.5%  UFH 6.7% | DVT or PE that resulted in hospitalisation or death with 120day of prophylaxis initiation  Bleeding that resulted in hospitalisation or death (including haemorrhagic stroke, GI, post procedure, Gynaecology, abdominal cavity, chest cavity, Coagulopathy, vascular access, haematoma or other e.g. orbital) | 2.7 vs 2.7 per 100 person years | RR=0.77 CI (0.49-1.22) p=0.04 for non-inferiority | 16.9 vs 17.2 bleeds per 100 patient years | RR= 0.98 (CI 0.78-1.23) p=0.02 for equivalence |

Subcutaneous (SC), once daily (od), twice daily (bd), three times daily (tds), Unfractionated heparin (UFH), Relative risk (RR), Venous thromboembolism (VTE), Deep vein thrombosis (DVT), Pulmonary embolism (PE), International Society on Thrombosis and Haemostasis (ISTH), Clinically relevant non-major bleeding (CRNMB), Gastrointestinal (GI)
