# Supplementary material for: A systematic review of the efficacy and safety of anticoagulants in advanced chronic kidney disease
Source: J Nephrol. 2022 Aug 25;35(8):2015–33. doi: 10.1007/s40620-022-01413-x (PMC9584987; doi:10.1007/s40620-022-01413-x)
Supplement: Supplementary file 4 — Supplementary file4 (DOCX 14 kb) [file 40620_2022_1413_MOESM4_ESM.docx]

Supplementary table 7

Studies of apixaban versus warfarin in CKD that include patients with VTE and AF

|  | Renal function | Number of patients | % patients with VTE, n | Apixaban Dosing in total population | VTE recurrence | Major bleeding | Baseline characteristics | Notes |
| --- | --- | --- | --- | --- | --- | --- | --- | --- |
| Herndon, 2020(76) | <30ml/min including dialysis | 111 | Apixaban 17% n=9  Warfarin 28% n=16 | 67% with VTE on 5mg bd | 0% versus 4% | 7% versus 14% p=0.362  For whole population | No details of VTE risk factors | Other indications included PAD and CAD |
| Stanton, 2017(75) | CrCl<25ml/min including PD/HD | 146 | 26% both groups n=19 | 61.6% on 2.5mg bd | None in either group | 9.6% apixaban vs 17.8%  For whole population | No details of VTE risk factors | Main indication was AF |
| Reed, 2018(73) | PD or HD | 124 | Apixaban 46% n=34  Warfarin 42% n=21 | 3 patients received 10mg bd loading dose  79.9% on 5mg bd | 4.4% vs 28.6%  P = .001 | 5.4% vs 22.0%  P =0 .01  For the whole study population | No details of VTE risk factors | Other indications were AF and VTE prophylaxis |
| Sarratt, 2017(78) | GFR<15ml/min | 160 | Apixaban 17.5% n=7  Warfarin  32.5% n=39 | 57.5% 2.5mg bd | Not reported | 0% versus 5.8% | No details of VTE risk factors. | Bleeding outcomes only |
| Schafer, 2018(74) | CrCl<30ml/min including dialysis | 604 | Apixaban 15.9% n=48  Warfarin 22.5%  n=68 | 52% 5mg bd | 1% versus 0.7% at 3months | 8.3% versus 9.9% at 3months | No details of VTE risk factors | 91% on 2.5mg bd were on incorrect dose |
| Hanni, 2020(77) | CrCl<25ml/min | 861 | Apixaban 15.6% n=20  Warfarin  47.7% n=350 | 57% 2.5mg bd | 5.5% versus 10% p=0.08  N.B composite thrombotic outcome of MI, DVT/PE, cardiac thrombus, stroke | 0.8 versus 1.6% p=ns | Previous VTE Apixaban 14.4% versus 32.1% | Multiple indications including metallic valve replacement (all on warfarin), cardiac thrombus |
